# Aerodynamics-assisted, efficient and scalable *kirigami* fog collectors

**DOI:** 10.1038/s41467-021-25764-4

**Published:** 2021-09-16

**Authors:** Jing Li, Ranjiangshang Ran, Haihuan Wang, Yuchen Wang, You Chen, Shichao Niu, Paulo E. Arratia, Shu Yang

**Affiliations:** 1 Department of Materials Science and Engineering, University of Pennsylvania, Philadelphia, PA 19104 USA; 2 Department of Mechanical Engineering and Applied Mechanics, University of Pennsylvania, Philadelphia, PA 19104 USA; 3 Key Laboratory of Bionic Engineering, Ministry of Education, Jilin University, Changchun, 130022 China

**Keywords:** Energy harvesting, Materials for energy and catalysis

## Abstract

To address the global water shortage crisis, one of the promising solutions is to collect freshwater from the environmental resources such as fog. However, the efficiency of conventional fog collectors remains low due to the viscous drag of fog-laden wind deflected around the collecting surface. Here, we show that the three-dimensional and centimetric *kirigami* structures can control the wind flow, forming quasi-stable counter-rotating vortices. The vortices regulate the trajectories of incoming fog clusters and eject extensive droplets to the substrate. As the characteristic structural length is increased to the size of vortices, we greatly reduce the dependence of fog collection on the structural delicacy. Together with gravity-directed gathering by the folds, the *kirigami* fog collector yields a collection efficiency of 16.1% at a low wind speed of 0.8 m/s and is robust against surface characteristics. The collection efficiency is maintained even on a 1 m^2^ collector in an outdoor setting.

## Abstract (web summary)

Water shortage not only occurs in arid regions, but also in humid area with little precipitation, despite abundant fog. Authors develop robust and scalable 3D centimetric kirigami structures to control wind flow and regulate the trajectories of incoming fog, yielding high fog collection efficiency.

## Introduction

**Fig. 1:**
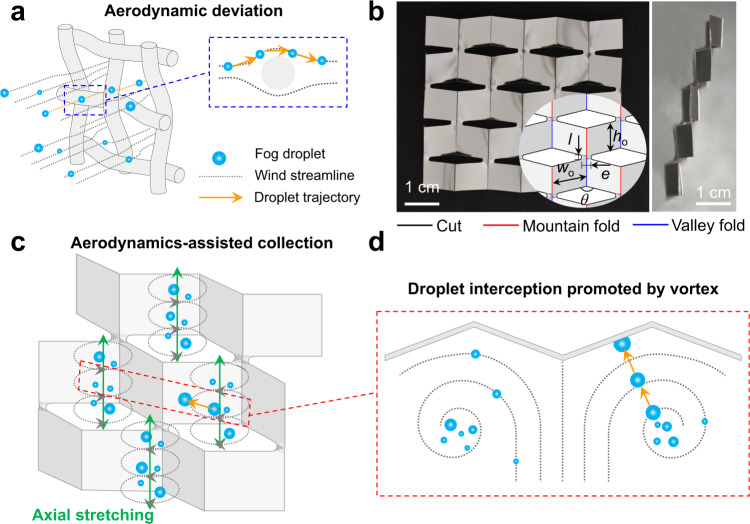
The design concept. **a** Schematic illustration of the aerodynamic deviation problem encountered by the conventional fog collecting mesh structures. The interception of fog droplets is highly impaired by the wind streamline deflected around the mesh fibers. **b** The front and side views of the cubic *kirigami* made from Al-coated PET. The insert image is the schematic showing of the cuts and folds. The height (*h*_o_) and width (*w*_o_) of cubic unit are set at 9 and 10 mm, respectively, while the width (*e*) and length (*l*) of the joint are 3 and 1 mm, respectively. The angle between two facets is defined as the folding angle $\theta$. **c** Conceptual drawing of the tube-like vortices penetrating through the openings. **d** Schematic image showing the trajectory of fog droplets rectified by the vortex. The incoming fog droplets can accumulate inside the vortex, where they grow larger through coalescence and are captured by the substrate efficiently.

Water shortage occurs not only in arid regions^1–3^, but also in humid regions with little precipitation despite abundant fog in such environments^4^. However, developing scalable and deployable structures that can efficiently and automatically collect freshwater from environments such as fog remains a major challenge^5–7^. First, due to the aerodynamic drag of wind deflected around the fog collectors (e.g. the mesh wires in Fig. 1a), the incoming fog droplets with low inertia closely follow the wind streamlines, going around the surface without being intercepted^5,7,8^. Second, tuning of surface structures^9–13^, chemistry/textures^6,14–20^, and impregnating lubricants^21,22^ facilitate the transport and drainage of fog droplets. However, these strategies rely on droplet-substrate interfacial effect and require fabrication of delicate structures much smaller than the droplet size at nano-/microscale, making them vulnerable to the physical damages and fouling in outdoor conditions for long-term applications.

Here, we address these challenges by tailoring the aerodynamics of fog-laden wind using *kirigami* structures, a more compliant variant of *origami* structures by leveraging both cutting and folding to shift a two-dimensional (2D) sheet to a three-dimensional (3D) structure independent of scales^23,24^. As seen in Fig. 1b, through mountain and valley folds, the perforated aluminum (Al)-coated PET sheet is transformed to cubic structure with satellite square facets separated by cuts and thus newly opened edges. Here, the height (*h*_o_) and width (*w*_o_) of each unit are set at 9 and 10 mm, respectively. Adjacent units are linked together through a joint with width (*e*) and length (*l*) of 3 and 1 mm, respectively. As shown in Supplementary Fig. 1, the curvature of the structure, or the folding angle (*θ*, see the definition in Fig. 1b), can be controlled precisely by lateral stretching. The fluid-structure (or wind-*kirigami* surface) interactions lead to the formation of tube-like vortices penetrating through openings due to axial stretching of the redirected air flow (Fig. 1c). These vortices normally have an inward flow toward their center. Hence, droplets can be attracted and accumulated near the vortex core, where they grow in size, gain inertia, and finally get ejected to the substrate (Fig. 1d). As a result, a much higher droplet interception efficiency can be obtained.

## Results

### Fast fog interception provided by *kirigami*-enabled vortices

**Fig. 2:**
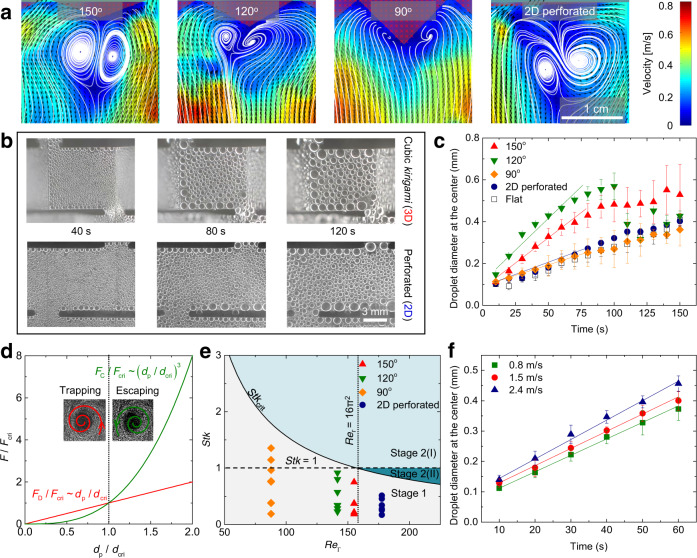
Origin of the fast fog capture provided by the *kirigami*-enabled vortices. **a** The velocity field of fog-laden air around the cubic *kirigami* surfaces with varying *θ*. A counter-rotating vortex pair is visualized by streamlines near the substrate, with their positions approaching the substrate as *θ* decreases. **b** Time-lapsed optical images revealing the droplet growth on one facet of cubic *kirigami* ($\theta$ = 150°) from the vertical view. **c** The variation of average diameters of droplets sitting at the center of facet as a function of time. **d** The variation of normalized drag force (*F*_D_/*F*_crit_) and centrifugal force (*F*_C_/*F*_crit_) as a function of the droplet diameter factor (*d*_p_/*d*_crit_). The critical droplet radius and force are ${d}_{{{{{{\rm{crit}}}}}}}=\frac{12\pi {\mu }_{{{{{{\rm{a}}}}}}}\delta }{{\varGamma }_{{{{{{\rm{o}}}}}}}\sqrt{{\rho }_{{{{{{\rm{a}}}}}}}{\rho }_{{{{{{\rm{w}}}}}}}/2}}\,$and ${F}_{{{{{{\rm{crit}}}}}}} \sim \frac{{\mu }_{a}^{3}r}{{\varGamma }_{{{{{{\rm{o}}}}}}}\delta {\rho }_{{{{{{\rm{a}}}}}}}\sqrt{{\rho }_{{{{{{\rm{a}}}}}}}{\rho }_{{{{{{\rm{w}}}}}}}/2}}\,$, respectively. **e** Phase diagram demonstrating different regimes of droplet motions. The data points show the measured ${Stk}$ and *Re*_г_ of the droplets trapped inside the vortex at various *θ*. All the experimental data lie in the gray region, and are in good agreement with our model: droplets at stage 1 have ${Stk}\le {St}{k}_{{{{{{\rm{crit}}}}}}}$. **f** The influence of wind speed on the droplet growth rate sitting at the center of the cubic *kirigami* ($\theta$ = 150°). Data in (**c**, **f**) are means ± s.d. of three measurements. In (**a**–**e**), the wind speed is kept at 0.8 m/s.

To explore the formation of vortices directed by *kirigami* geometries, we visualize the fog flow near the substrate using particle image velocimetry (PIV, see Supplementary Fig. 2). The wind speed is set to ${u}_{{{{{{\rm{\infty }}}}}}}=$ 0.8 m/s, the typical light wind speed in a calm day, corresponding to a Reynolds number (*Re*) of ~500; *Re*, the relative importance of fluid inertia to viscous forces, is defined as: ${Re}={u}_{{{\infty }}}{w}_{{{{{{\rm{o}}}}}}}/{\nu }_{{{{{{\rm{a}}}}}}}$, where ${\nu }_{a}$ is the air kinematic viscosity. As shown in Fig. 2a, we find a counter-rotating vortex pair formed around the mountain fold, which creates additional stagnation regions in front of the curved substrate. These stagnation regions can not only attract the incoming fog droplets but modify their trajectories near the substrate. As *θ* decreases, both vortex size $\delta$ and circulation ${\varGamma }_{{{{{{\rm{o}}}}}}}$ reduce (Supplementary Fig. 3). In addition, at *θ* = 150° and 120°, the vortices flutter back and forth (i.e., unsteady motion) around the fold at frequencies of 6 and 4.5 Hz, respectively (Supplementary Video 1 and Supplementary Fig. 4), during which the droplets accumulating inside the vortices can deposit onto the substrate, due to droplet inertia.

To verify the feasibility of using such quasi-stable counter-rotating vortices to promote fog interception, we measure the droplet growth dynamics on one facet of cubic *kirigami* under the same flow condition (Method and Supplementary Fig. 5). To quantify the droplet size, we dip coat the surface with amorphous perfluoroether (Cytop^®^), which has a water contact angle of 105.3 ± 1.1° and a low contact angle hysteresis (the difference between the advancing and receding contact angles) of 9.2 ± 1.3°. As shown in Fig. 2b, which presents the sample snapshots (*θ* = 150°) visualized from the vertical view (see Supplementary Fig. 6 for the front view), the droplet diameter at the center of cubic facet is much larger than those on the perforated surface without folding (Supplementary Video 2). We plot the time evolution of the average diameter of visible droplets (diameter >80 μm) under various *θ* in Fig. 2c. At *θ* = 150° and 120°, droplets grow to an average diameter ~0.5 mm after 60 s, following a weak exponential function. The average growth rates of droplets, or the slopes of the plots, are 5.42 × 10^−3^ mm/s (*θ* = 150°) and 6.76 × 10^−3^ mm/s (*θ* = 120°), respectively, which decreases as *θ* deviates from this range. The fast droplet growth rate manifested on the cubic *kirigami* is in sharp contrast to that on a perforated 2D sheet (1.91 × 10^−3^ mm/s; see the snapshots in Supplementary Fig. 7). Such an effective capture and growth of droplets can also be evidenced by the average coalescence frequencies after 60 s (Supplementary Fig. 8a), showing a similar trend as *θ* changes. As a result, extensive droplets with diameters >500 μm appear on the cubic *kirigami* with *θ* = 120° and 150°, whereas droplets of smaller diameter <300 μm dominate on the 2D perforated surface (Supplementary Fig. 8b). Since the size of the cubic unit is four orders of magnitude larger than that of the incoming fog droplets and there are no specially fabricated nano-/micro-structures on the surface, we believe that the quick droplet deposition and growth must arise from the rectified aerodynamics of wind alone.

To gain further insights into how vortical wind affects droplet behaviors, we develop a simplified model that describes the flow field of each vortex using Burgers vortex flow equations^25,26^ (Methods). We limit the analysis to steady symmetric vortices resembling Fig. 2a, and neglect the interactions between droplets. The radial motion of a droplet inside a Burgers vortex is dominated by two forces^27^: an air drag force ${F}_{{{{{{\rm{D}}}}}}}$ and a centrifugal force ${F}_{{{{{{\rm{C}}}}}}}$, pointing inward and outward the vortex (Fig. 2d), respectively. The drag force is ${F}_{{{{{{\rm{D}}}}}}}=3\pi {d}_{{{{{{\rm{p}}}}}}}{\mu }_{{{{{{\rm{a}}}}}}}({\dot{r}-u}_{{{{{{\rm{r}}}}}}})$, where ${d}_{{{{{{\rm{p}}}}}}}$ is the droplet diameter,$\,{\mu }_{{{{{{\rm{a}}}}}}}$ is the viscosity of air, *r* is the radial position of the droplet, $\dot{r}$ and ${u}_{{{{{{\rm{r}}}}}}}$ are the radial velocities of the droplet and vortex, respectively. The centrifugal force is ${F}_{{{{{{\rm{C}}}}}}}=\pi {d}_{p}^{3}{\rho }_{w}{\varOmega }^{2}r/6$, where ${\rho }_{w}$ is the density of water, $\varOmega ={\varGamma }_{{{{{{\rm{o}}}}}}}/\left(4\pi {\delta }^{2}\right)$ is the angular velocity. By linearizing the tangential velocity of droplet in the vicinity of vortex center as: ${u}_{{{{{{\rm{\theta }}}}}}}\left(r\right)=\varOmega r+O\left({r}^{2}\right)$, the motion of droplet at *r* can be obtained as (see Method):

1 $$\frac{d}{{dt}}\left[\begin{array}{c}r\\ \dot{r}\end{array}\right]=\left[\begin{array}{cc}0 & 1\\ {\varOmega }^{2}-\alpha /{t}_{{{{{{\rm{p}}}}}}} & -1/{t}_{{{{{{\rm{p}}}}}}}\end{array}\right]\left[\begin{array}{c}r\\ \dot{r}\end{array}\right],$$

where ${t}_{{{{{{\rm{p}}}}}}}={\rho }_{{{{{{\rm{w}}}}}}}{d}_{{{{{{\rm{p}}}}}}}^{2}/(18{\mu }_{{{{{{\rm{a}}}}}}})$, is the Stokes time, $\alpha$ denotes the strain rate. Equation (1) becomes critically stable when ${\varOmega }^{2}-\alpha /{t}_{{{{{{\rm{p}}}}}}}=0$. Note that this stability criterion is not relevant to the initial condition of the incoming fog droplets. Finally, we can obtain the stability criterion in the form of critical particle diameter as:

2 $${d}_{{{{{{\rm{crit}}}}}}}=\frac{12\pi {\mu }_{{{{{{\rm{a}}}}}}}\delta }{{\varGamma }_{{{{{{\rm{o}}}}}}}\sqrt{{\rho }_{{{{{{\rm{a}}}}}}}{\rho }_{{{{{{\rm{w}}}}}}}/2}}.$$

We note that ${d}_{{{{{{\rm{crit}}}}}}}$ depends on flow parameters $\delta$ and ${\varGamma }_{{{{{{\rm{o}}}}}}}$, which can be related to the geometric parameter $\theta$ through empirical relations obtained from PIV experiments (see Supplementary Fig. 3). As shown in Fig. 2d, ${F}_{{{{{{\rm{C}}}}}}}$ grows at a much faster pace than ${F}_{{{{{{\rm{D}}}}}}}$ for ${d}_{{{{{{\rm{p}}}}}}} \; > \; {d}_{{{{{{\rm{crit}}}}}}}$, under which the droplet will be ejected out of the vortex.

The critical droplet size can be put into dimensionless form: ${St}{k}_{{{{{{\rm{crit}}}}}}}=16{\pi }^{2}/{{Re}}_{\Gamma }$, by defining a Stokes number (${Stk}={t}_{{{{{{\rm{p}}}}}}}{\varGamma }_{{{{{{\rm{o}}}}}}}/{\delta }^{2}$) and a vortex Reynolds number (${{Re}}_{\Gamma }={\varGamma }_{{{{{{\rm{o}}}}}}}{\rho }_{a}/{\mu }_{{{{{{\rm{a}}}}}}}$). Depending on ${Stk}$, the droplet behaviors can be categorized into two stages (Fig. 2e and Supplementary Fig. 9). Stage 1: For the incoming small fog droplets with ${Stk}\;\le\; {St}{k}_{{{{{{\rm{crit}}}}}}}$, they move toward the vortex core, where they accumulate and grow larger through coalescence. Stage 2: As droplets get large enough so that ${Stk} \; > \; {St}{k}_{{{{{{\rm{crit}}}}}}}$, they are ejected outward from the vortex. Depending on ${St}{k}_{{{{{{\rm{crit}}}}}}}$, two conditions will occur: (I) ${Stk} \; > \; {St}{k}_{{{{{{\rm{crit}}}}}}} \; > \; 1$, the ejected droplets deviate from the wind streamlines, and thus they can be captured by the substrate efficiently. (II) $1\ge {Stk} \; > \; {St}{k}_{{{{{{\rm{crit}}}}}}}$, the ejected droplets can be carried away by the wind, leading to lower fog interception. To check the reliability of the model, we measure the ${Stk}$ of droplets trapped inside the vortex of ${{Re}}_{\Gamma }$ (see Methods for measurement). As shown in Fig. 2e, all the data points lie in the gray region, in good accordance with the model, which predicts that the droplets at Stage 1 have ${Stk}\le {St}{k}_{{{{{{\rm{crit}}}}}}}$. One would like to operate in Stage 2(I) (the light green region in Fig. 2e), corresponding to *θ* < 150°. Within this regime, *θ* = 120° and 150° yield the smallest ${St}{k}_{{{{{{\rm{crit}}}}}}}$, which explains the highest fog growth rate observed in experiments.

As the vortex-assisted fog collection mechanism addresses the aerodynamic deviation limitation at low *Stk*^8^, it alleviates the dependence of fog capture on the wind speed. As plotted in Fig. 2f, the growth rate of droplets on cubic *kirigami* with *θ* of 150° is improved steadily from 5.42 × 10^−3^ to 6.41 × 10^−3^ mm/s as the wind speed is increased from 0.8 to 2.4 m/s. In contrast, the collection performance of mesh/wire collectors can be easily affected by the small fluctuation in wind speed^28^. Moreover, the aerodynamics-assisted droplet capture highly reduces the dependence of fog interception on the structure delicacy. As shown in Supplementary Fig. 10, at a wind speed of 0.8 m/s, enhanced droplet growth rates can be sustained on the *kirigami* surface if the unit size (*w*_o_) is smaller than 2 cm. Such a threshold length scale of structure further increases under higher wind speeds.

Meanwhile, the extensive cut edges, another feature of our *kirigami* structures, also play an important role in the fog interception. Owing to the large hysteresis, the droplets sitting at the edges absorb surrounding ones, forming chains of large droplets along the edges. As plotted in Supplementary Fig. 11, the average diameter of droplets increases continuously and the average growth rate within the first 60 s reaches 11.10 × 10^−3^ mm/s, ~6.4 times that on surface without cuts (1.74 × 10^−3^ mm/s). The growth rate is nearly independent of *θ*, except *θ* = 120° shows a somewhat larger rate.

### Aerodynamics-assisted pyramidal *kirigami* fog collector

**Fig. 3:**
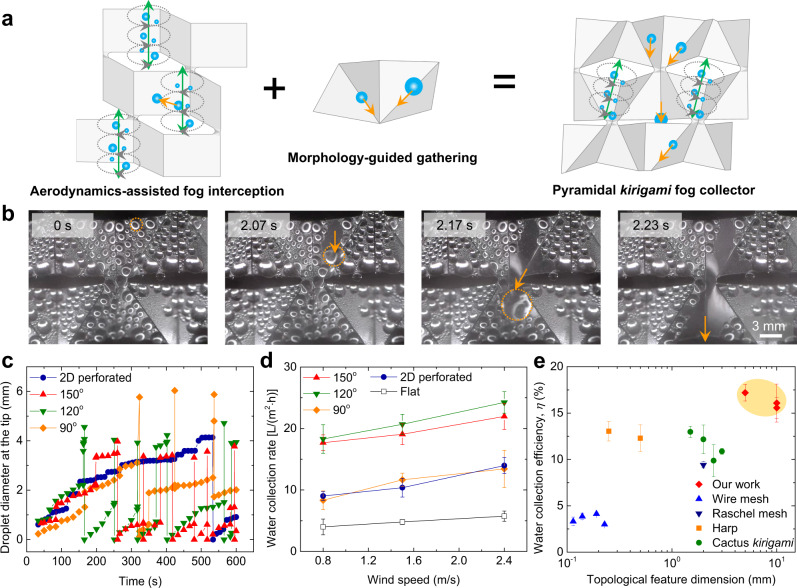
The efficient dripping and collection of fog droplets on the pyramidal *kirigami*. **a** The design of pyramidal *kirigami* structure for highly efficient fog collection. It combines the aerodynamics-assisted fog interception induced by the geometric curvature with the directed droplet gathering arising from the asymmetric morphology. **b** Selected snapshots of the transport and dripping of droplets rectified by the valley folds. **c** Time evolution of the droplet diameter sitting at the bottom tip of concave pyramid. The sudden drop of droplet diameter indicates the dripping of droplets. **d** The variation of stabilized water collection rate as a function of wind speed and folding angle. **e** The comparison of the collection efficiency (*η*) of various fog collectors. Here, $\theta$ = 150°. Data in (**d**, **e**) are means ± s.d. of five measurements.

The aerodynamics-assisted fog capture allows us to design highly efficient fog collectors through the combination with asymmetric surface morphology. By adding one more fold along the diagonal line of each facet and keeping *h*_o_ and *w*_o_ the same, the cubic *kirigami* structure is transformed to a pyramidal shape with morphological asymmetry in the vertical direction (Supplementary Fig. 12). By controlling $\theta$, we maintain the geometric curvature and openings required by the formation of vortices along the folds. In this regard, it allows us to decouple the fog harvesting process into two separate stages (Fig. 3a): an array of curvature-induced vortices to promote the interception of incoming droplets, and the asymmetric and connected folds to directionally gather the deposited droplets. Thus, we can bypass the trade-off between the efficient droplet deposition and fast removal encountered by the conventional fog collectors^29,30^. As the droplet sitting at the edge grows large enough, it falls in an avalanche-like behavior, grabbing substantial droplets from the center region of each facet (Fig. 3b and Supplementary Video 3). Because of the anisotropic energy threshold rendered by the bumped valley fold^31^ (Supplementary Fig. 13), the falling droplet is guided along the valley channel, and finally drips from the bottom tip of the concave pyramid. As seen in Fig. 3c, owing to the conjunction of the effective fog interception and directed transport, droplets on *kirigami* surface with *θ* = 120° depart from the surface much earlier than others. Importantly, the concave-convex pyramids translate the interception and dripping of fog droplets into different planes, naturally avoiding the clogging problems in mesh systems^5,7^ (Supplementary Video 4). Consequently, the pyramidal *kirigami* samples can sustain a high and stable dripping frequency even after 2 h (Supplementary Fig. 14; over 40 counts/h for each bottom tip).

Figure 3d compares the overall water collection rate under different folding angles and wind speeds. The highest collection rates, occurring at *θ* = 120° and 150°, are 18.3 L/(m^2^ h) and 17.7 L/(m^2^ h) under a wind speed of 0.8 m/s, respectively, which gradually rise to 24.2 L/(m^2^ h) (*θ* = 120°) and 21.9 L/(m h) (*θ* = 150°), respectively, as the wind speed is increased to 2.4 m/s. Note that the water collection rate is calculated on the basis of the actual surface area. Moreover, the collection efficiency^7,32^, defined as *η* = *v*_co_/*v*_in_ can reach ~16.1% at a low wind speed of 0.8 m/s (Supplementary Fig. 15). Here, *v*_co_ and *v*_in_ are the water collection rate and the water delivery rate, respectively. We compare the efficiency of our aerodynamics-assisted *kirigami* fog collector with other state-of-the-art fog collectors, including wire mesh^5,13^, harp^13^, cactus *kirigami*^11^, and Raschel mesh^5^, under the same experimental conditions in Fig. 3e (see Supplementary Fig. 16 for specific parameters and selected snapshots and Supplementary Fig. 17 for water collection rate). Note that all the fog collectors are coated with Cytop^®^ to eliminate the influence of surface interfacial properties on the conventional designs. The collection efficiency on pyramid *kirigami* is much higher even though the topological unit size is 10–100 times larger than the structure size of conventional fog collectors.

**Fig. 4:**
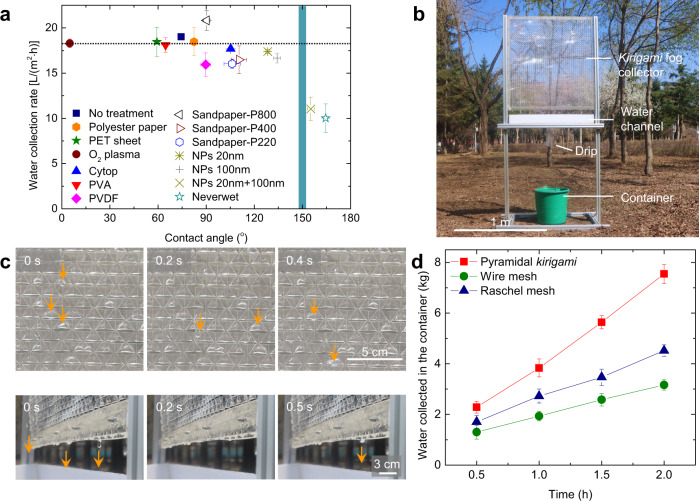
The stability and scalability of the pyramid *kirigami* fog collector. **a** The variation of water collection rates (under a wind speed of 0.8 m/s) on the pyramidal *kirigami* samples made from different materials and with different surface treatments as a function of water contact angles. **b** A photo of the large-scale fog collection system. The pyramidal *kirigami* with unit width of 1 cm is cut and folded from a 1 ×1 m PET sheet. **c** The selected snapshot images showing the transport (top panel) and dripping (bottom panel) of fog droplets on *kirigami* collector in the outdoor testing. The experiments were conducted during April 15–19, 2021 in Changchun, China with the environmental temperatures ranging of 12–19 °C, and the wind speed and humidity near the sample as 2.5 ± 0.8 m/s and 98 ± 1.7%, respectively. **d** The weight of water collected by the meter-scale *kirigami* fog collector as the function of time. Fog collections on the wire mesh (wire diameter: 150 μm, open area: 30%) and Raschel mesh (wire width: 2 mm, shade rate: 50–60%) were tested under the same environmental conditions for comparison. Data in (**a**, **d**) are means ± s.d. of five measurements. Here, $\theta$ = 150°.

Since the aerodynamics-assisted fog harvesting is a geometry-enabled effect, the collection performance is robust against mechanical abrasion and fouling in an outdoor setting, and can be applied to various affordable and *non-permeable* materials. As evidenced by Fig. 4a, regardless of surface treatment and choice of materials (Supplementary Note 1, Supplementary Table 1 and Supplementary Fig. 18, 19), the pyramidal *kirigami* can sustain an average water collection rate of 17.5 L/(m^2^ h) as long as the apparent water contact angle is below 150^o^. Note that when the apparent water contact angle is >150°, the droplets undergo coalescence-induced random jumping, lowering water collection rate [~10.5 L/(m^2^ h)], which is consistent with literature^33^.

### Outdoor testing of meter-scale *kirigami* fog collector

Importantly, the overall size of pyramidal *kirigami* collector can be scaled-up, e.g. from a 1 × 1 m PET sheet (Supplementary Fig. 20) (Method). To exploit the feasibility of scaling up *kirigami* fog collectors in meter-scale in practical settings, we build a fog collection system using aluminum profiles (Fig. 4b), and test its collection efficiency under an outdoor industrial humidifier in Changchun, China (43.88°N, 125.35°E). The data are collected during April 15–19, 2021 with the environmental temperatures of 12–19 °C, and the wind speed and humidity near the sample is controlled at 2.5 ± 0.8 m/s and 98 ± 1.7 %, respectively. As seen in Fig. 4c, a frequent water dripping is obtained bypassing the clogging problem at the same time. In contrast, on wire mesh and Raschel mesh samples, the clogging problem occur immediately once the fog droplet grows larger (Supplementary Fig. 21). Over a period of 2 h, the average fog collection rate of pyramidal *kirigami* is 3.51 kg/h (corresponding to an efficiency of 16.6% under the fog delivery rate of 21.1 kg/h), which is 2.71 and 1.82 times those from the wire mesh and Raschel mesh, respectively, under the same testing condition (Fig. 4d). *Kirigami* structures can be further modularized to create larger scale collectors and deployed flexibly at different locations at an estimated price of $35– $175 depending on the choice of materials, fabrication cost and the installation locations (Supplementary Fig. 22).

## Discussion

In summary, we develop centimetric and 3D *kirigami* structures that generate the rectified vortical wind and regulate its interactions with the fog droplets laden inside, which substantially elevates the fog collection efficiency without delicate structures. Importantly, the aerodynamics-assisted fog collection mechanism demonstrated here is mainly governed by the global geometry. Therefore, it is applicable to a wide variety of substrates, and feasible at meter scale. From a border perspective, the aerodynamics-assisted *kirigami* fog collection will not only enhance the society’s water and energy resilience, but also reduce the cost and dependence on infrastructure.

## Methods

### Materials

Al-coated PET sheet (#48-5F-1M-13, .005”, CS Hyde Company), ethanol (195 proof, Decon), 2-propanol (Fisher Scientific), N, N-dimethylformamide (Fisher Scientific), Cytop^®^ CTL-109 AE (AGC Chemicals), Fluorinert™ FC-3283 (TMC Industries Inc.), poly(vinyl alcohol) (PVA, average Mw ~125,000, Sigma-Aldrich), poly(vinylidene fluoride) (PVDF, average Mw ~534,000, Sigma-Aldrich), P800, P400 and P220 sandpaper sheets (3M Co.), SiO_2_ nanoparticles (NPs) with different sizes (Nissan Chemicals), (tridecafluoro-1,1,2,2-tetrahydrooctyl) trichlorosilane (SIT8174.0, Gelest Inc.), NeverWet Multi-Surface (Rust Oleum), Polyester paper (0.008”, Durilla Synthetics), PET sheet (0.006”, eMigoo) are purchased and used as received.

### Sample preparation

Various cubic and pyramidal *kirigami* samples are fabricated from Al-coated PET sheet with thickness of 127 μm. Specifically, the Al-coated PET sheet is cut through along the black solid lines over a length of 17 mm using Cricut^®^ cutting machine (Explore air 2), leaving a joint with width and length of 3 and 1 mm, respectively. To ensure the mechanical robustness of the structure, the edge of joint that connects the adjacent *kirigami* units, is designed to be round (diameter: 1 mm) in order to more evenly distribute the stress during folding^34^. After that, the surface is scored with a smaller force along the red and blue lines, leaving channels with depth of 100 μm. By alternating the mountain and valley folds along the red and blue lines, respectively, the flat sheet is folded into 3D and conformable *kirigami* structures, with the length (*h*_o_) and width (*w*_o_) of *kirigami* unit being 9 and 10 mm, respectively. All the samples are completely washed with ethanol and 2-propanol sequentially, followed by dip-coating with a diluted Cytop^®^ CTL-109AE solution (mixed with solvent, Fluorinert™ FC-3283, at a mass ratio of 1:50) at a speed of 3 mm/s. Finally, all the samples are baked at 100 °C for 20 min to completely remove the Fluorinert™ FC-3283. The treated surfaces are characterized by water goniometer (Ramé-hart instrument Co.) with a water contact angle of 105.3 ± 1.1° and a contact angle hysteresis (the difference between advancing and receding contact angles) of 9.2 ± 1.3°, averaged over 5 measurements.

### The control of the folding angles

The folding angle (*θ*) of the cubic or pyramidal *kirigami* structure is controlled by confining the positions of joints. In particular, for the cubic configuration, the spacings between joints in the horizontal (2*w*) and vertical ($h+l$) directions can be related to *θ* as $2w=2{w}_{{{{{{\rm{o}}}}}}}\;{\sin }\left(\theta /2\right)$ and $h+l={h}_{o}+l$, respectively. For the pyramidal configuration, $2w=2{w}_{{{{{{\rm{o}}}}}}}\;{\sin }\left(\theta /2\right)$, and $h+l=\sqrt{{{h}_{{{{{{\rm{o}}}}}}}}^{2}+{\left[{w}_{{{{{{\rm{o}}}}}}}{\cos }\left(\theta /2\right)\right]}^{2}}+l$.

### The wind tunnel setup

A customized fog-laden wind tunnel is designed to mimic the fog condition in the real world. As schematically shown in Supplementary Figs. 2 and 5, fog clusters consisting of suspended droplets with an average radius of 5 μm are generated by an ultrasonic humidifier (Air-O-Swiss AOS 7135), which delivers a water volume (*V*_total_) of 0.36 L/h. The fog droplets are guided into an acrylic cylinder tube (US Plastic Corp.) with length and inner diameter of 15 and 6.35 cm, respectively, through a hole (diameter, 4 cm) drilled 2 cm away from the inlet of the tube. This setup is to straighten fog droplets and filter out the turbulence in the fog spray before reaching the *kirigami* collector. The speed of the fog flowing inside the tube is controlled and adjusted by the USB cooling fan (AC Infinity MULTIFAN S1, 80 mm USB Fan) settled at the inlet of the tube. Meanwhile, a 3D printed ABS straightener with a hexagonal hole array (side length 3.5 mm, Supplementary Fig. 2), which is pre-treated with commercial water repellent coating (NeverWet Multi-Surface, Rust Oleum), is fixed at the outlet of the tube to rectify the fog cluster into a uniform and laminar flow. The tube is tilted at an angle of 15° to ensure the visualization of the samples. To avoid the occurrence of condensation that may affect the fog collection result, we make the size of testing sample (unit size of 1 cm and overall width of 5 cm) smaller than the diameter of wind tunnel. As a result, the relative humidity near the sample is stabilized at 99.9% (445580, ExtechTM). The temperatures of the substrate, the water and the sample holders are measured the same as the room temperature (20.5 ± 0.8 °C; TMD-50 K-type thermocouple thermometer, Amprobe).

### PIV measurement

During the measurement, the cubic *kirigami* surfaces are sprayed with commercial waterproof paint (Montana, Black) to prevent their reflection. The fog-laden laminar wind is illuminated by the green laser sheet (517 nm, 50 mW, BioRay), with a direction parallel with the coming fog and a focus at the center of the cubic unit (Supplementary Fig. 2). The flow filed of fog-laden wind near the *kirigami* structure is recorded by a high-speed camera (Fastcam SA1.1, Photron) equipped with a lens (AF-D, f/2.8, 80–200 mm, Nikon) and an extension tube (DG 20 mm, Nikon) under a frame rate of 1500 fps. The direction of high-speed camera is set to be vertical to the incoming fog.

### Theoretical modeling of droplet movement inside the vortex

We model the vortices as the “Burgers vortex”^25^, the tangential, radial and axial velocity components of which can be expressed as:

1 $${u}_{\theta }\left(r\right)=\frac{{\varGamma }_{{{{{{\rm{o}}}}}}}}{2\pi r}\left[1-{\exp }\left(-\frac{{r}^{2}}{2{\delta }^{2}}\right)\right],$$

2 $${u}_{r}=-\alpha r,$$

3 $${u}_{z}=2\alpha z,$$

Here, ${\varGamma }_{{{{{{\rm{o}}}}}}}$ is the vortex circulation, $\delta =\sqrt{{\nu }_{a}/\alpha }$ is the vortex size, ${\nu }_{a}$ is the kinetic viscosity of air, and $\alpha$ is the strain rate. It can be shown that Eqs. (1–3) are the steady state solutions of Navier–Stokes equation and vorticity equation^26,35^. The Froude number of the coming fog is calculated as: $F{r}_{{{{{{\rm{p}}}}}}}={u}_{\infty }/\sqrt{g{d}_{{{{{{\rm{o}}}}}}}}\approx 110\gg 1$, where *g* = 9.8 m/s^2^, and ${d}_{{{{{{\rm{o}}}}}}}\approx 5$ μm is the average size of incoming droplets. Thus, the gravity of droplets can be ignored. Since the droplet Reynolds number is $R{e}_{{{{{{\rm{p}}}}}}}={u}_{\infty }{d}_{{{{{{\rm{o}}}}}}}/{\nu }_{{{{{{\rm{a}}}}}}}\ll 1$, the drag force ${F}_{{{{{{\rm{D}}}}}}}$ can be modeled by Stokes law, and the motion of droplet at the radial position *r* can be obtained as:

4 $$m\frac{d\dot{r}}{{dt}}={F}_{{{{{{\rm{C}}}}}}}-{F}_{{{{{{\rm{D}}}}}}}=m{\varOmega }^{2}r+3\pi {d}_{{{{{{\rm{p}}}}}}}{\mu }_{{{{{{\rm{a}}}}}}}\left({u}_{r}-\dot{r}\right),$$

where $\varOmega ={\varGamma }_{{{{{{\rm{o}}}}}}}/\left(4\pi {\delta }^{2}\right)$ is the angular velocity of the droplet,$\,{\mu }_{{{{{{\rm{a}}}}}}}$ is the dynamic viscosity of air, $m={\rho }_{{{{{{\rm{w}}}}}}}\pi {d}_{{{{{{\rm{p}}}}}}}^{3}/6$ is the mass of the droplet, ${\rho }_{{{{{{\rm{w}}}}}}}$ is the density of water, and ${d}_{{{{{{\rm{p}}}}}}}$ is the diameter of the droplet. By introducing the Stokes time of fog particle: ${t}_{{{{{{\rm{p}}}}}}}={\rho }_{{{{{{\rm{w}}}}}}}{d}_{{{{{{\rm{p}}}}}}}^{2}/(18{\mu }_{{{{{{\rm{a}}}}}}})$, we get:

5 $$\frac{d\dot{r}}{{dt}}={\varOmega }^{2}r+\frac{{u}_{{{{{{\rm{r}}}}}}}-\dot{r}}{{t}_{{{{{{\rm{p}}}}}}}}.$$

We linearize Eq. (1) in the vicinity of vortex center as: ${u}_{{{{{{\rm{\theta }}}}}}}\left(r\right)=\frac{1}{2}{\omega }_{{{{{{\rm{o}}}}}}}r+{{{{{\rm{O}}}}}}\left({r}^{2}\right)$, with ${\omega }_{{{{{{\rm{o}}}}}}}=2\varOmega$ representing the vorticity at the center of vortex. Then, Eq. (5) can be recast into a linear dynamical system:

6 $$\frac{d}{{dt}}\left[\begin{array}{c}r\\ \dot{r}\end{array}\right]=\left[\begin{array}{cc}0 & 1\\ {\omega }_{{{{{{\rm{o}}}}}}}^{2}/4-\alpha /{t}_{{{{{{\rm{p}}}}}}} & -1/{t}_{{{{{{\rm{p}}}}}}}\end{array}\right]\left[\begin{array}{c}r\\ \dot{r}\end{array}\right].$$

The linear stability of Eq. (6) determines the movement of the fog particle, which is controlled by the two eigenvalues of the state-transition matrix:

7 $${\lambda }_{1,2}=\frac{-b\pm \sqrt{{b}^{2}-4c}}{2},$$

where $b=1/{t}_{{{{{{\rm{p}}}}}}}$, $c={\omega }_{{{{{{\rm{o}}}}}}}^{2}/4-\alpha /{t}_{{{{{{\rm{p}}}}}}}$. To maintain a stable particle trajectory, the real parts of both eigenvalues need to be non-positive, which leads to the stability criterion: $c\ge 0$. Note that this stability criterion does not depend on the initial condition of the coming fog droplets. The droplet trajectory is critically stable when $c=0$, or Stokes time equals to ${t}_{{{{{{\rm{crit}}}}}}}=4\alpha /{\omega }_{{{{{{\rm{o}}}}}}}^{2}$. Finally, we can obtain the stability criterion in the form of a critical particle diameter as:

8 $${d}_{{{{{{\rm{crit}}}}}}}=\frac{12\pi {\mu }_{{{{{{\rm{a}}}}}}}\delta }{{\varGamma }_{{{{{{\rm{o}}}}}}}\sqrt{{\rho }_{{{{{{\rm{a}}}}}}}{\rho }_{{{{{{\rm{w}}}}}}}/2}},$$

Equation (8) corresponds to a critical vortex stokes number of: ${St}{k}_{{{{{{\rm{crit}}}}}}}=16{\pi }^{2}/{{Re}}_{\Gamma }$.

To prove the reliability of the model, we measure the *Stk* of droplets moving inside the vortex. Specifically, the droplets trapped inside the vortex are chosen randomly, and their sizes are estimated using Crocker & Grier’s tracking algorithm^36^. The experimental data show good agreements with the model (see Fig. 2e).

We note, however, there are additional factors that can affect the motion of droplets, including: (1) Droplet coalescence. As trapped droplets coalesce near the vortex core, the released surface energy will convert to momentum in random directions. (2) Interaction with substrate. At smaller *θ* (i.e. 90°), the vortices approach the substrate (Fig. 2a). Owing to the interaction with the substrate^37,38^, the vortices become unsteady (Supplementary Fig. 4), leading to the compromised fog deposition. (3) Vortex fluttering. At desired *θ* (120° or 150°), the vortices flutter back and forth around the fold, bringing a large amount of fog droplets to the substrate directly, whereas no flutter motion is observed at *θ* = 90° and 180° (Supplementary Fig. 4a). Nevertheless, the model provides quantitative guidance on the effects of wind turbulence engendered by global geometric curvature on the effective interception of fog droplets.

### The visualization of fog collection process

The fog collection dynamics is monitored by Nikon camera (D5600) mounted with a micro lens (AF-S VR Micro-Nikkor 105 mm f/2.8 G IF-ED) under a frame rate of 60 fps. In the vertical view, the camera is settled vertically to one cubic facet, while in the front view, the camera is set towards the mountain fold.

### Large-scale outdoor testing

The large-scale *kirigami* structure is fabricated by laser cutting and scoring (PLS 4.75) the PET sheets with thickness of 150 μm. After being folded into 150°, the sample is embedded into an acrylic frame. During the outdoor testing, the industrial humidifier (LX-20MC08B, Zhengcheng Company) delivering fog droplets with average radius of 7 μm at water volume (*V*_total_) of 21.1 L/h is used to mimic the fog condition in the practical settings. The spout diameter of the humidifier is 0.8 m. The fog collection test is conducted in Changchun, China from April 15–19, 2021, during which the weather temperature and humidity are 12–19 °C and 16–25%, respectively. During the test, the wind speed and humidity near the sample are measured at 2.5 ± 0.8 m/s and 98 ± 1.7%, respectively. The water collection rate is obtained by measuring the weight of container every 30 min (Supplementary Fig. 21).

## Supplementary information

Supplementary Information

Description of Additional Supplementary Files

Supplementary Movie 1

Supplementary Movie 2

Supplementary Movie 3

Supplementary Movie 4

Supplementary Movie 5

## Data Availability

Data supporting the findings of this study are available from corresponding author S.Y. (shuyang@seas.upenn.edu) upon reasonable request.
