# Supplementary material for: Aerodynamics-assisted, efficient and scalable kirigami fog collectors
Source: Nat Commun. 2021 Sep 16;12:5484. doi: 10.1038/s41467-021-25764-4 (PMC8445985; doi:10.1038/s41467-021-25764-4)
Supplement: Supplementary file 1 — Supplementary Information [file 41467_2021_25764_MOESM1_ESM.pdf]

Supplementary Information:

Aerodynamics-assisted, efficient and scalable *kirigami* fog collectors

Jing Li[1], Ranjiangshang Ran[2], Haihuan Wang[1], Yuchen Wang[1], You Chen[3], Shichao Niu[3], Paulo E. Arratia[2], Shu Yang[1,*]

[1]Department of Materials Science and Engineering, University of Pennsylvania, Philadelphia, PA 19104, USA

[2]Department of Mechanical Engineering and Applied Mechanics, University of Pennsylvania, Philadelphia, PA 19104, USA

[3]Key Laboratory of Bionic Engineering, Ministry of Education, Jilin University, Changchun 130022, China

Corresponding author: shuyang@seas.upenn.edu (S.Y.)

Supplementary Note 1

The surfaces of pyramidal *kirigami* structures fabricated from aluminum (Al)-coated PET sheet (#48-5F-1M-13, .005", CS Hyde Company) are treated chemically and physically to elucidate their robustness and stability against damages and fouling. Extended Data Table 1 summarizes the resulting surface wettability.

Materials**:** Ethanol (195 proof, Decon), 2-propanol (Fisher Scientific), N, N-dimethylformamide (Fisher Scientific), Cytop CTL-109 AE (AGC Chemicals), Fluorinert™ FC-3283 (TMC Industries Inc.), poly(vinyl alcohol) (PVA, average Mw ~ 125,000, Sigma-Aldrich), poly(vinylidene fluoride) (PVDF, average Mw ~ 534,000, Sigma-Aldrich), P800, P400 and P220 sandpaper sheets (3M Co.), SiO_2 nanoparticles (NPs) with different sizes (Nissan Chemicals), (tridecafluoro-1,1,2,2-tetrahydrooctyl) trichlorosilane (SIT8174.0, Gelest Inc.), NeverWet Multi-Surface (Rust Oleum), Polyester paper (.008", Durilla Synthetics), PET sheet (.009", eMigoo) are purchased and used as received.

Surface treatment 1**:** To explore the effect of surface chemistry on the water harvesting performance, the pyramidal *kirigami* surfaces are treated by various polymers. First, the *kirigami* samples after being folded are cleaned by ethanol and 2-propanol. They are then treated by O_2 plasma for 5 min, followed by dip-coating of different polymers, including Cytop (0.18 wt % in Fluorinert™ FC-3283), PVA (1 wt % in deionized water), PVDF (5 wt % in N, N-dimethylformamide) at a speed of 3 mm/s. Finally, all the samples are baked at 100 ^{o}C for 20 min to remove the residual solvent.

Surface treatment 2**:** To mimic the physical damages such as stretches in the outdoor environment, the cut Al-coated PET sheets are roughened by P800, P400 and P220

sandpapers, respectively, after which they are folded into 150°. Finally, the pyramidal *kirigami* surfaces are ultrasonically cleaned in ethanol for 30 min to remove any residual debris. Note that the Al coating on the PET sheets can be partially removed by the sandpaper, and thus the measured contact angles in Extended Data Table 1 are higher than those on the pristine surface.

***Surface treatment 3*:** To mimic fouling, SiO_2 NPs of different sizes are employed to modify the surface of the pyramidal *kirigami*. Specifically, SiO_2 NPs with diameter of 20 nm and 100 nm are cleaned and dried following our previous paper[1], after which they are well-dispersed in ethanol at a concentration of 10 wt%. For the dual-scale SiO_2 NPs suspension, 20 nm and 100 nm NPs are added to ethanol at a concentration of 6 wt%, respectively[2], and stirred for 5h. Different NPs solutions are dip-coated onto the folded pyramidal *kirigami* at a rate of 1 mm/s. Finally, the samples are dried and treated with (tridecafluoro-1,1,2,2-tetrahydrooctyl)trichlorosilane (SIT8174.0) through the chemical vapor deposition. In addition to the coating of SiO_2 NPs, the cleaned surface is directly sprayed with commercial superhydrophobic coating, NeverWet Multi-Surface, as a supplementation.

Supplementary Table 1 The apparent water contact angles (CAs) and contact angle hysteresis (CAH) on the pyramidal *kirigami* structures made of different materials and after various surface treatments.

**Treatment method**	**CA measurement**	**Advancing CA (θ_a)**	**Receding CA (θ_r)**	**CAH= θ_a - θ_r**
No treatment	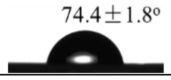	86.4±1.8°	27.9±2.3°	58.5±2.1°
O_2 plasma-5 min	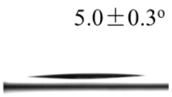	5.2±0.9°	Nil	Nil
Cytop (0.18 wt %)	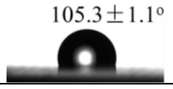	110.0±1.5°	100.8±1.1°	9.2±1.3°
PVA (1 wt %)	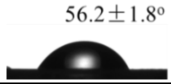	78.9±2.1°	14.8±1.9°	64.1±2.0°
PVDF (2.5 wt %)	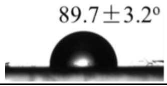	96.8±1.0°	61.1±0.6°	35.7±0.8°
Sandpaper-P800	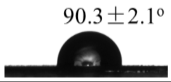	96.1±2.6°	7.9±0.8°	88.2±1.7°
Sandpaper-P400	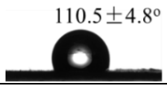	116.6±2.3°	5.6±0.3°	111.0±1.3°
Sandpaper-P220	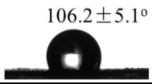	106.7±2.8°	6.9±1.4°	99.8±2.1°
SiO_2 NPs: 20 nm (10 wt %)	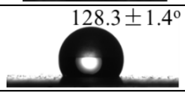	129.1±2.5°	68.6±1.3°	60.5±1.9°
SiO_2 NPs: 100 nm (10 wt %)	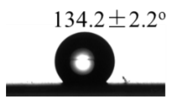	150.1±1.1°	75.2±1.1°	74.9±1.1°
SiO_2 NPs: 20 nm (6 wt %) + 100 nm (6 wt %)	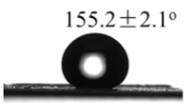	157.7±0.8°	152.3±0.6°	5.4±0.7°
NeverWet Multi-Surface	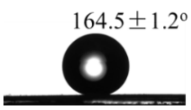	165.9±1.1°	161.8±0.7°	4.1±0.9°
Polyester paper	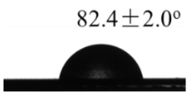	96.3±2.1°	13.6±1.7°	82.7±1.9°
PET sheet	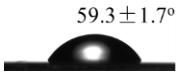	66.9±1.3°	23.2±2.6°	43.7±2.0

Supplementary Figures

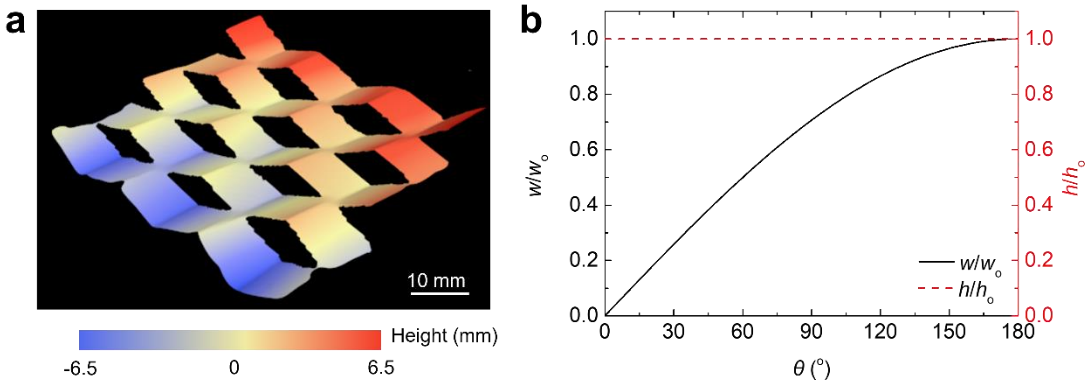

Supplementary Fig. 1| Topography of the cubic *kirigami* structure. a, The 3D scanning image showing the cubic *kirigami* structure. **b**, The tunability of cubic *kirigami* structure in both the horizontal (w/w_o) and vertical (h/h_o) directions. The surface can be opened and closed completely in the horizontal direction.

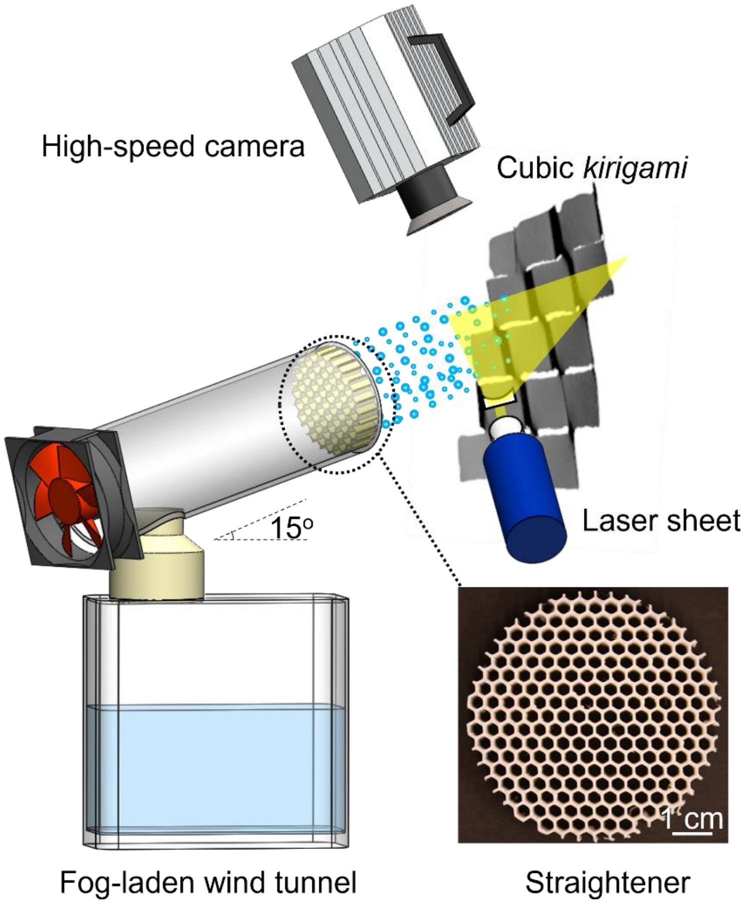

Supplementary Fig. 2| Experimental setup of PIV measurement. The laminar fog-laden air flow is generated by the wind tunnel system consisting of humidifier, tube, 3D printed ABS straightener and wind fan. During the test, the wind-tunnel is tilted by an angle of 15° for the later droplet visualization. The laser sheet is focused on the center of one cubic unit, with the direction coincidence with the coming fog. The high-speed camera is set to be vertical to the incoming fog flow.

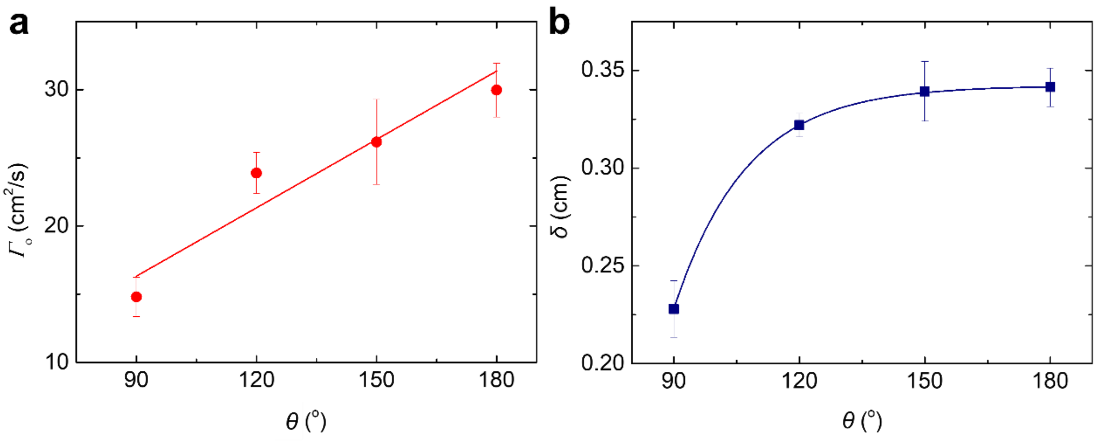

Supplementary Fig. 3| The characterization of vortex at the symmetric state on the cubic *kirigami* surface shown in Fig. 2a. **a**, Average vortex circulation ($\Gamma_{\rm o}$) as a function of the folding angle (θ), showing an increasing trend in $\Gamma_{\rm o}$ as θ increases. The linear fitting (solid red line) for $\Gamma_{\rm o}$ is $\Gamma_{\rm o} = (1.3 + 0.17\theta)\ \mathrm{cm}^2/\mathrm{s}$. **b**, Vortex size (δ) as a function of θ, where δ first increases with θ, then saturates. The fit (solid blue curve) is an exponential function: $\delta = 0.34\{1 - \exp[-0.06(\theta - 68.44°)]\}$ cm. Based on this empirical relation, at a small θ where the vortex approaches the substrate, a minor variation in θ can greatly affect the vortex size. Note that the 2D perforated surface corresponds to θ of 180° in the plots. All the error bars are the s.d. of three measurements.

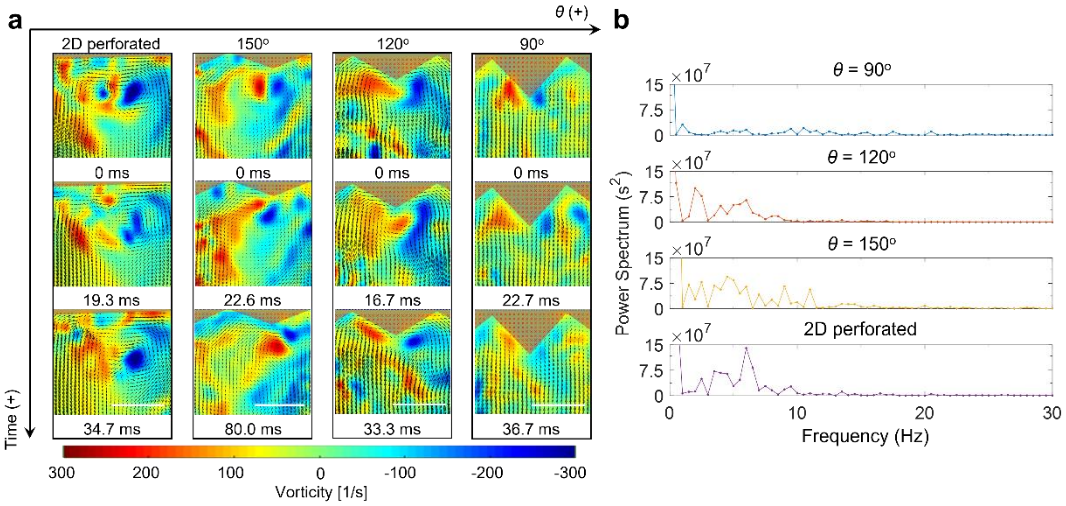

Supplementary Fig. 4| The characterization of vortex pair in front of the cubic *kirigami* surface (unit width: 1 cm) with varying *θ*. a, The vorticity field. The coherent vortices rotate in the opposite directions. At θ of 120° and 150°, the cores of vortices flutter back and forth around the mountain fold, bringing extensive fog droplets towards the surfaces. Such a flutter behavior disappears at θ = 90° and on a 2D perforated surface. Scale bars: 1 cm. **b**, The power spectra of the average vorticity. By taking the fast Fourier transformation (FFT), the frequencies of vortices at θ = 120°, 150° and 180° are obtained as 2.5 Hz, 4.5 Hz and 6 Hz, respectively. However, at θ = 90°, the vortices exist unsteadily owing to the interactions with the substrate and thus there is no dominating frequency.

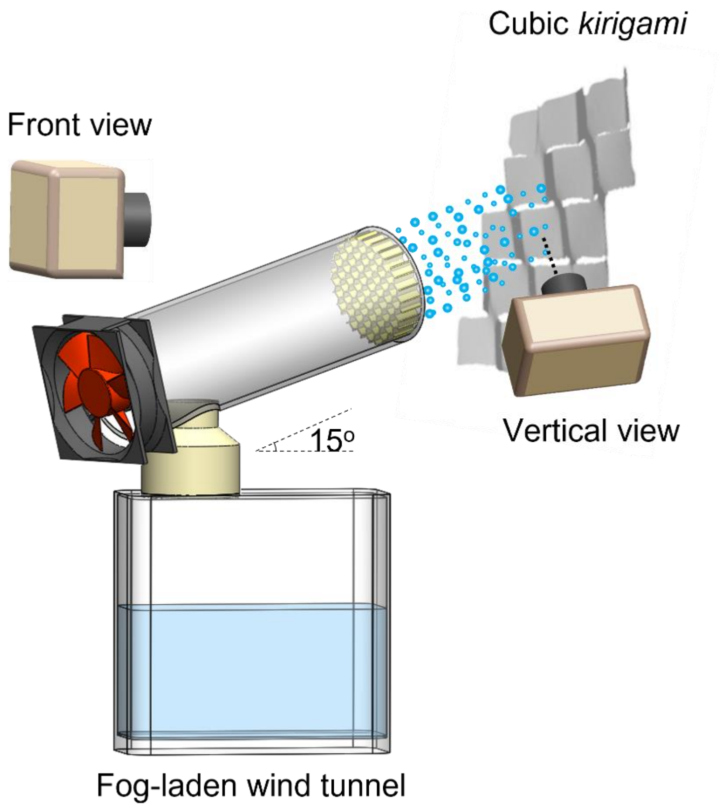

Supplementary Fig. 5| Schematic of the experimental setup for the fog visualization. During the test, the wind-tunnel is tilted by an angle of 15° for the visualization of droplet behavior from different directions.

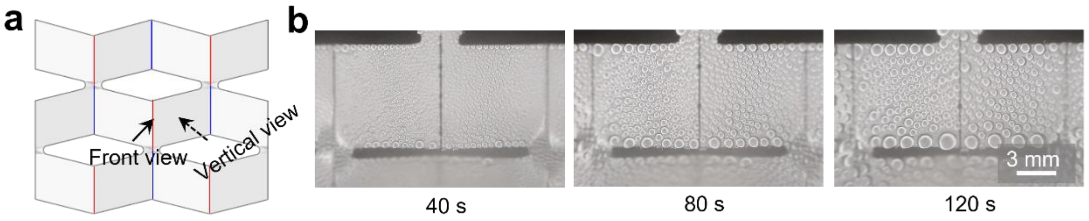

Supplementary Fig. 6| a, Illustration of two directions to visualize fog behaviors on a cubic *kirigami* structure. In the front view, the camera is set towards the mountain fold, whereas in the vertical view, the camera is arranged vertically to one cubic facet. **b**, Selected snapshot photos demonstrating the fast capture and growth of fog droplets on the cubic *kirigami* structure (θ=150°) from the front view. Here, the width of *kirigami* unit (w_o) and the wind speed are 1 cm and 0.8 m/s, respectively.

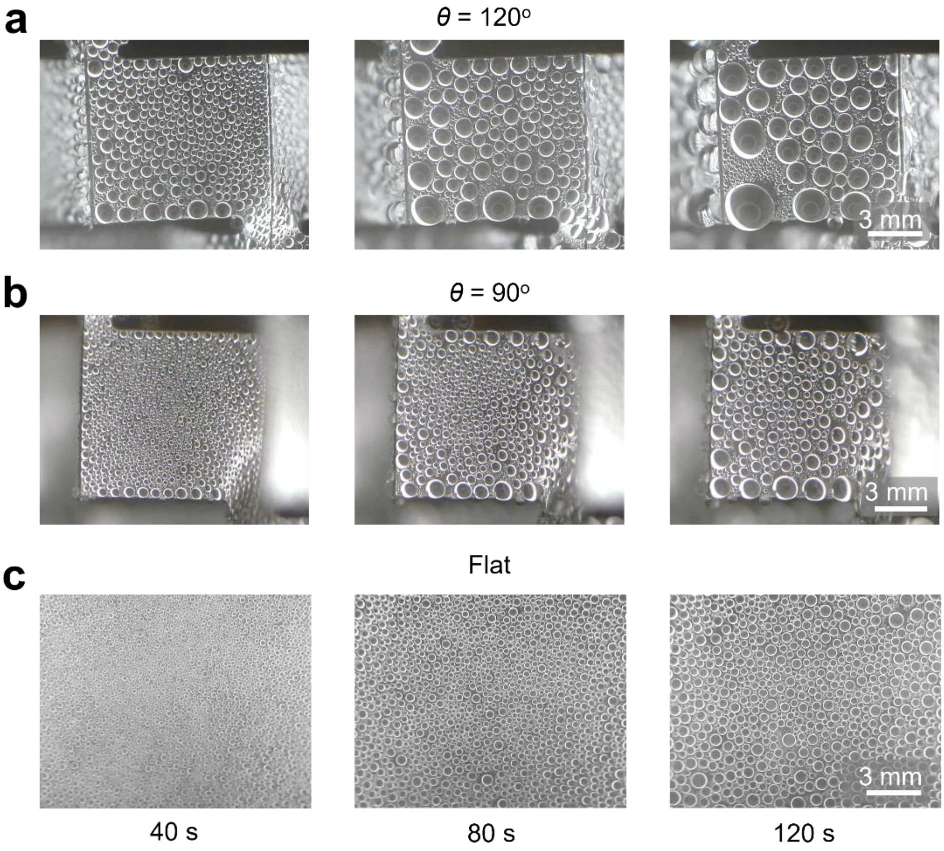

Supplementary Fig. 7| Selected snapshot pictures showing the capture and growth of fog on cubic *kirigami* surfaces with different θ. a, θ = 120º. **b**, θ = 90º. **c**, Flat surface without cuts and folds. The width of *kirigami* unit (w_o) is 1 cm and the wind speed is 0.8 m/s.

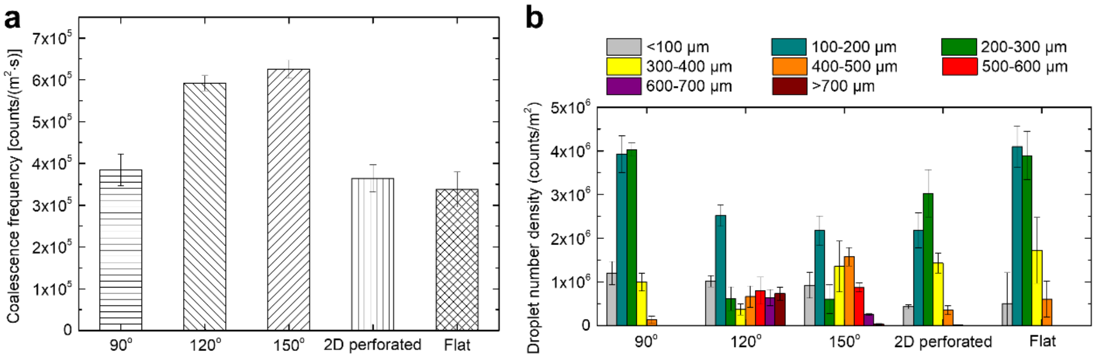

Supplementary Fig. 8| Characterization of droplet growth dynamics on the center of the cubic *kirigami* (w_0: 1 cm) with varied θ at the early stage. **a**, The average coalescence frequency of droplets within the first 60 s. Here, the coalescence frequency is counted by comparing two adjacent snapshots at a frame rate of 30 fps. **b**, The size distribution of water droplets at 60 s on the center of the cubic *kirigami* with different θ *vs*. the 2D perforated surface and flat surface. All the error bars are the s.d. of five measurements.

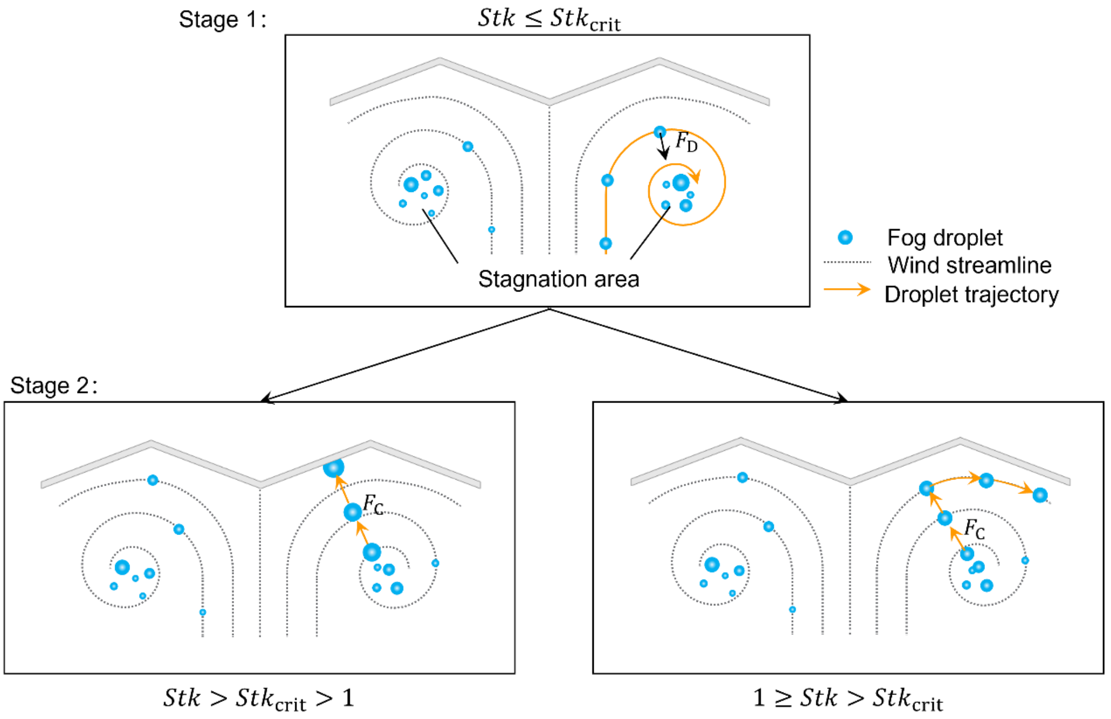

Supplementary Fig. 9| Schematic illustration of different stages of the interception of fog droplets. Stage 1: For the incoming fog droplets that are often small in sizes and thus have a low Stk, they migrate along the streamline of vortex and are attracted towards the vortex core due to Stokes drag. Stage 2: As the droplets grow large enough near the vortex core through coalescence ($Stk > Stk_{crit}$), they are ejected out of the vortex by centrifugal force. (I) Under the condition that $Stk > Stk_{crit} > 1$, the ejected droplets can be caught by the surface efficiently. (II) Under the condition that $1 \geq Stk > Stk_{crit}$, the ejected droplets can be affected by the streamline of the deflected air around the substrate, and are brought away before they reach the substrate.

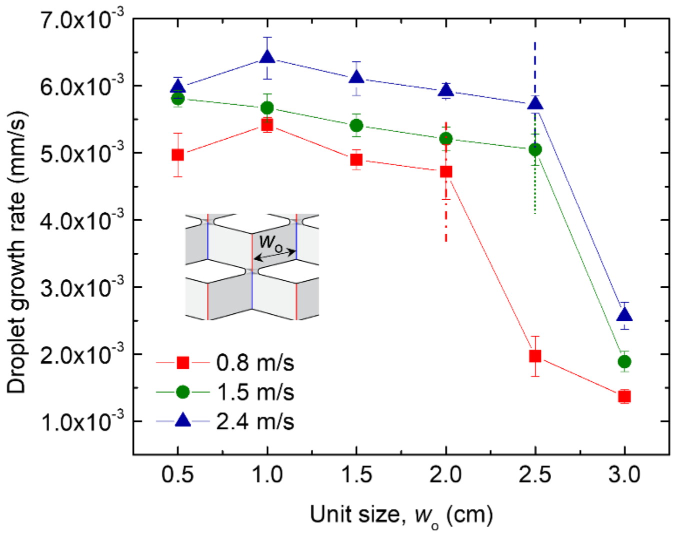

Supplementary Fig. 10| The growth rate of droplet on cubic *kirigami* in the first 60 s as a function of unit size (w_o). Here, $\theta = 150^o$. All the error bars are the s.d. of three measurements.

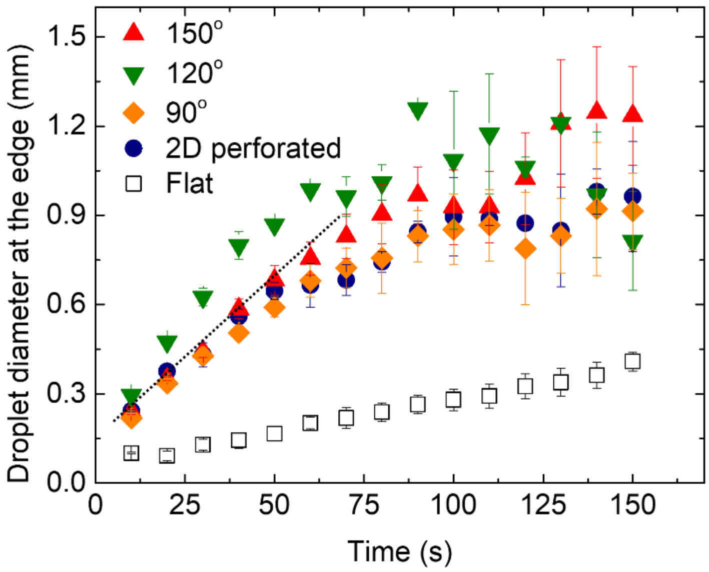

Supplementary Fig. 11| The variation of average diameters of droplets sitting at the edge of cubic *kirigami* as a function of time. The average growth rate, or the slope of dotted line, are calculated at 11.10×10^{-3} mm/s. Data are means ± s.d. of three measurements. w_o and the wind speed are kept at 1 cm and 0.8 m/s, respectively.

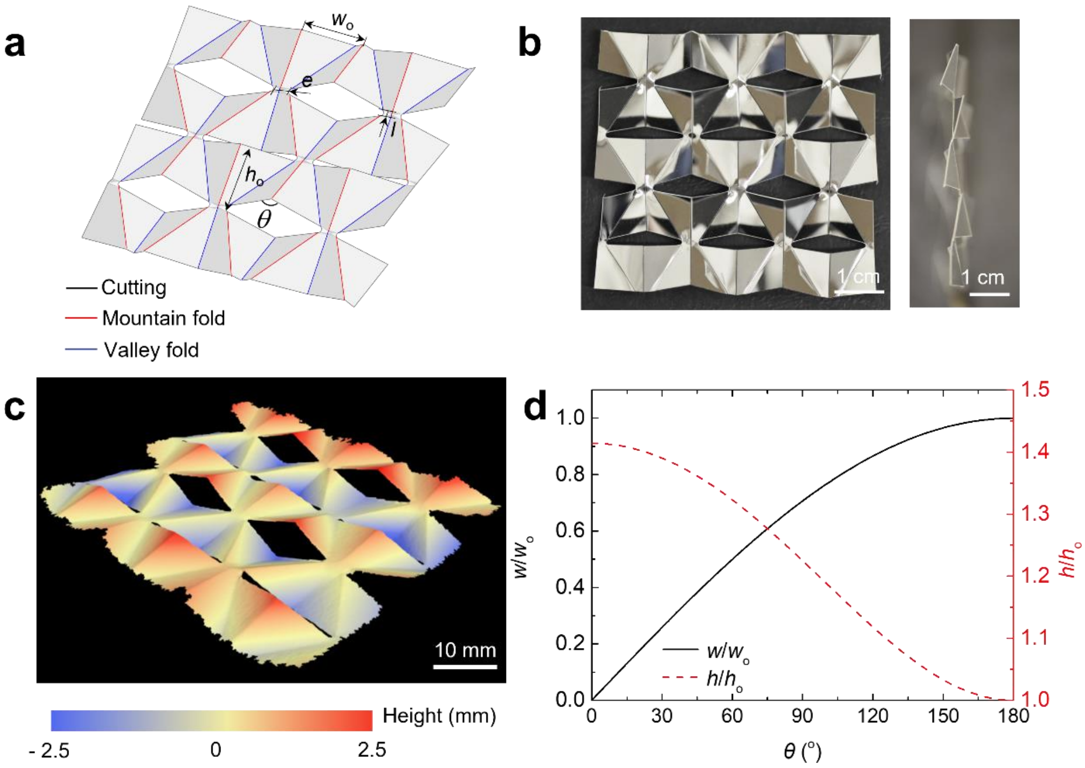

Supplementary Fig. 12| The geometric characterization of pyramidal *kirigami* surface. a, Schematic illustration of the pyramidal *kirigami* surface with the cutting edges in black lines, the mountain and valley folds in red and blue lines, respectively. The height (h_o) and width (w_o) of pyramidal unit as well as the width (e) and length (l) of joint are kept the same as the cubic configuration: $h_o = 0.9$ cm, $w_o = 1$, $e = 3$ mm and $l = 1$ mm. **b**, The front-view and side-view images of the pyramidal *kirigami* fabricated from an Al-coated PET sheet based on design in (**a**). **c**, 3D scanning image of (**b**). **d**, Deformability of the pyramidal *kirigami* in the vertical and horizontal directions, which is defined as the ratio of the unit width (w) and height (h) after folding to those before folding (w_o and h_o). The curvature between adjacent faces, or the folding angle θ can be easily controlled through either lateral or vertical stretching.

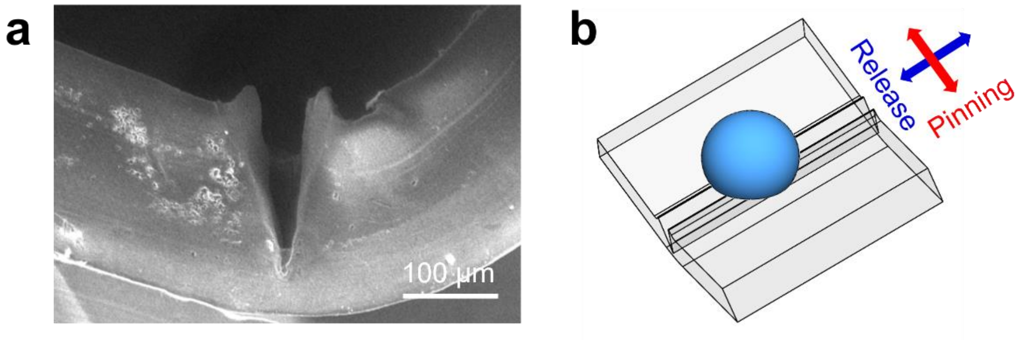

Supplementary Fig. 13| The bumped valley channel. a, SEM images of the localized morphologies at the valley folds. Owing to the deformation of Al-coated PET sheet after folding, the scored channel is transformed into bumped tracks. **b**, Rectified by the asymmetric morphology of the bumped valley fold, the droplet is highly mobile only along the track.

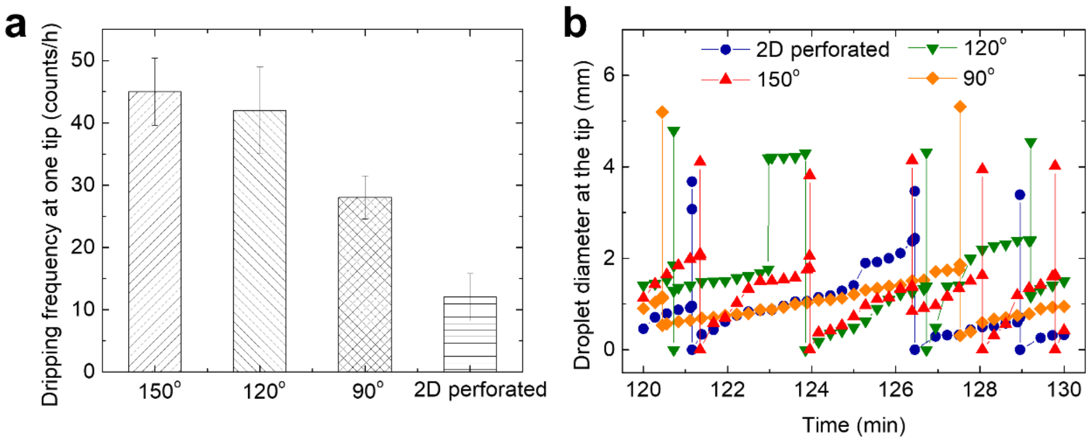

Supplementary Fig. 14| Long time testing. **a**, The stabilized dripping frequency at one bottom tip of concave pyramid measured over 2 h. Data are means ± s.d. of three measurements. **b**, The variation of droplet diameter at the bottom tip after 2 h. The *kirigami* surface maintains a stable and frequent water dripping over long period, confirm its durability. The wind speed and unit width (w_o) are 0.8 m/s and 1 cm, respectively.

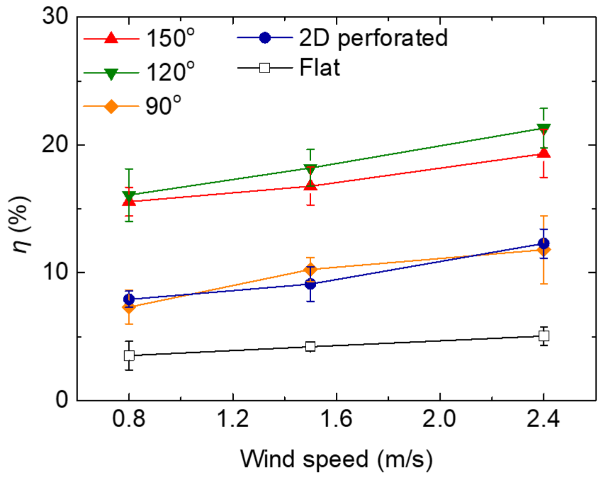

Supplementary Fig. 15| The water collection efficiency on pyramidal *kirigami* (w_o = 1 cm) as a function of wind speed and θ. Here, the water collection efficiency $\eta = v_{co} / v_{in}$, where v_{co} is obtained from Fig. 3d, v_{in} can be estimated as $v_{in} = V_{total} / A$, with v_{co} and v_{in} being the water collection rate and the water delivery rate, respectively, V_{total} and A being the delivery rate of the humidifier and the inner section area of the spray tube (Methods), respectively. All the error bars are the s.d. of five measurements.

a

Wire mesh	Wire diameter (mm)	Open aera
	0.114	30 %
	0.140	30 %
	0.191	31 %
	0.229	30 %
Harp	Wire diameter (mm)	Pitch to wire diameter
	0.25	2
	0.5	2
Cactus *kirigami*	Spine-to-spine spacing (mm)	Spine width (mm): 1<br>Spine length (mm): 5
	1.5	
	2	
	2.5	
	3	
Raschel mesh	Wire width (mm)	Shade rate
	2	50 %-60 %

b

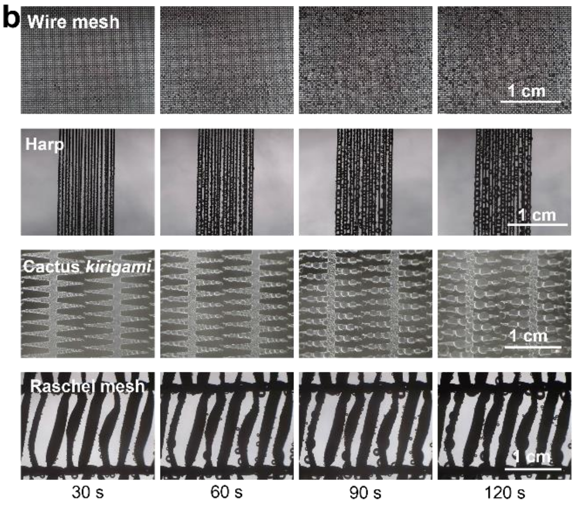

Supplementary Fig. 16| The performance of the conventional fog collectors. a, Parameters of the state-of-the-art fog collectors including wire mesh[3,4], harp[4], cactus *kirigami*[5] and Raschel mesh[3]. **b**, Selected snapshots showing the fog collection on wire mesh (wire diameter: 0.140 mm, open aera: 30%), harp (wire diameter: 0.25 mm, pitch to wire diameter: 2), cactus *kirigami* (spine-to-spine spacing: 1.5 mm) and Raschel mesh (wire width: 2 mm, shade rate 50%-60%). Note that all the snapshot images are captured using our fog systems as described in Method, and the wind speed is controlled at 0.8 m/s.

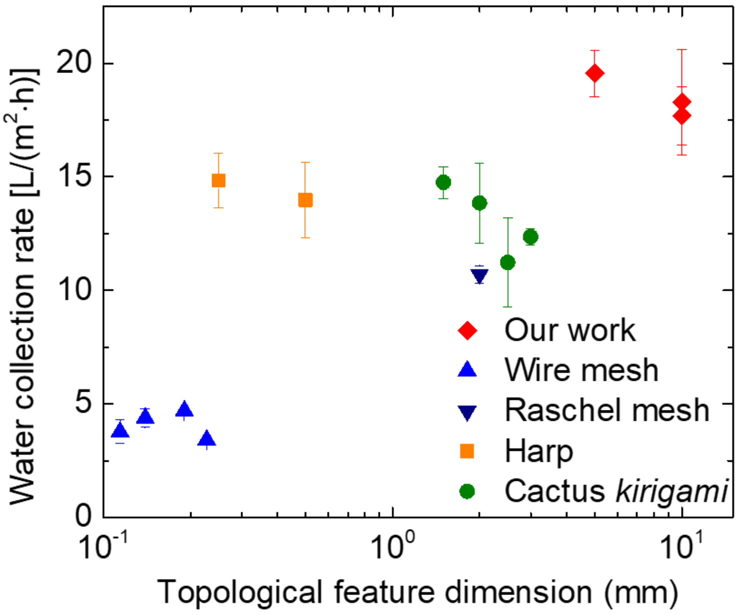

Supplementary Fig. 17| Comparison of the fog collection rate of various fog collectors[3-5] elaborated in Supplementary Fig. 16. Note that all the data are obtained using our fog systems under a wind speed of 0.8 m/s, which are different from the values reported in Ref. 3-5 owing to different experimental conditions. All the error bars are the s.d. of three measurements.

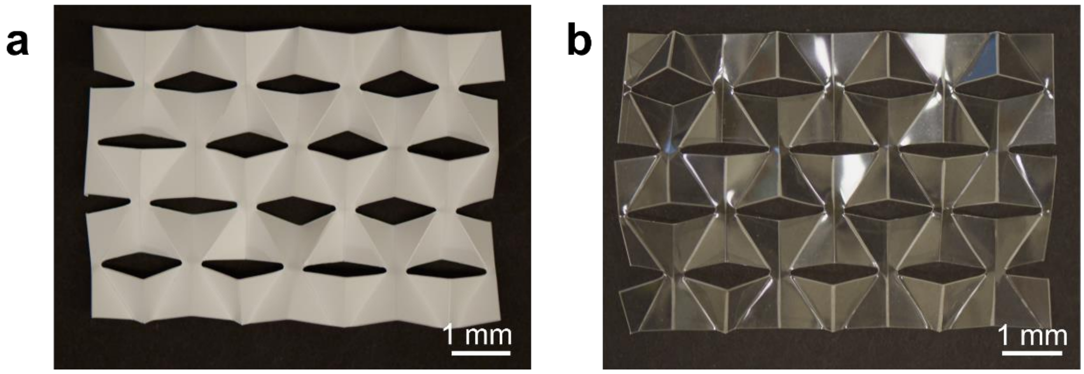

Supplementary Fig. 18| Pyramidal *kirigami* surfaces made of different materials.

a, Polyester coated paper. **b**, PET sheet.

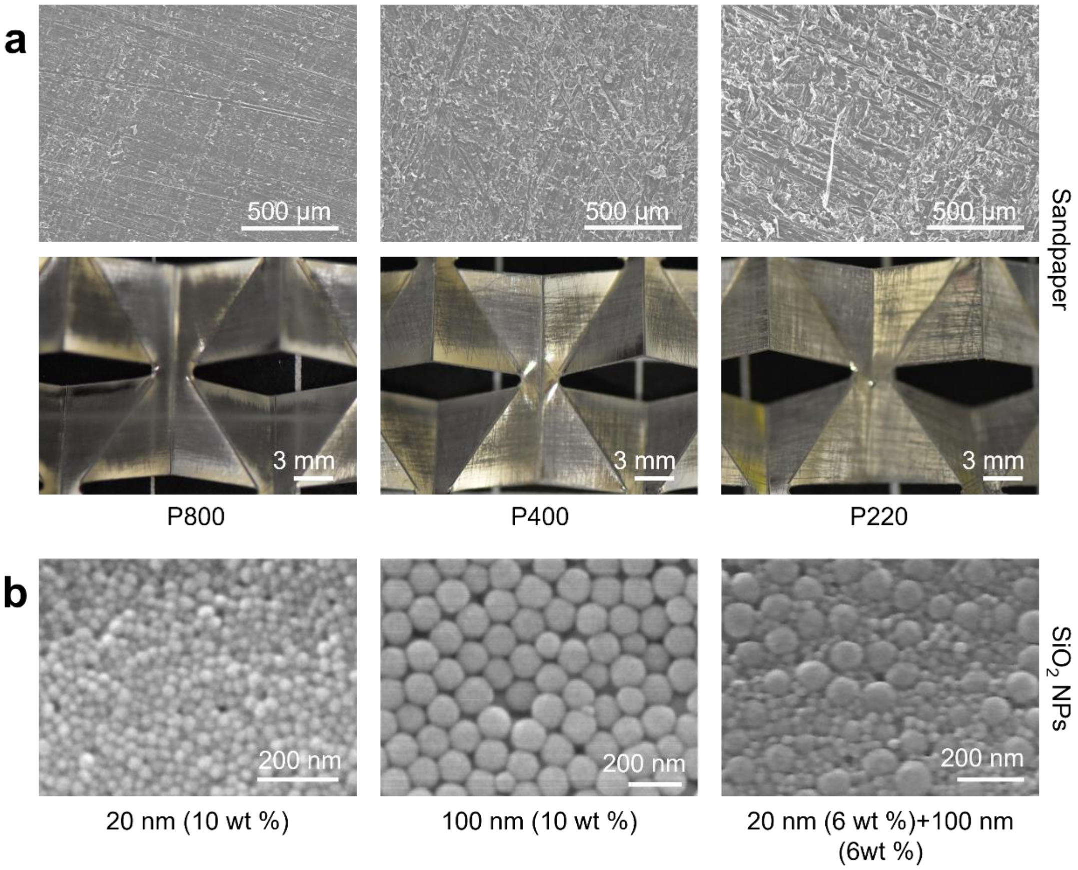

Supplementary Fig. 19| Morphologies of the pyramidal *kirigami* structures made from Al-coated PET after various surface treatments. **a**, SEM (top) and the corresponding optical images (bottom) of pyramidal surfaces scratched by sandpaper with roughness of P800, P400 and P220, respectively. **b**, SEM images of surfaces after being dip-coated with SiO_2 nanoparticles with different sizes.

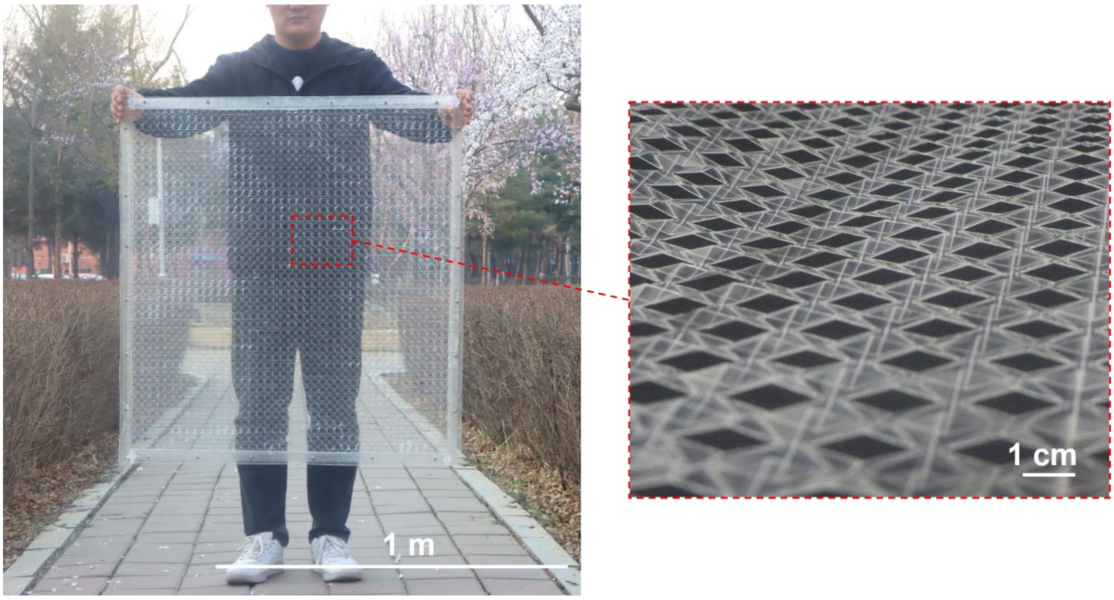

Supplementary Fig. 20| The meter-scaled pyramidal *kirigami* sample. The unit width w_o is 1 cm. The sample is cut from a 1 m × 1 m PET sheet (thickness of 150 μm) with θ = 150°.

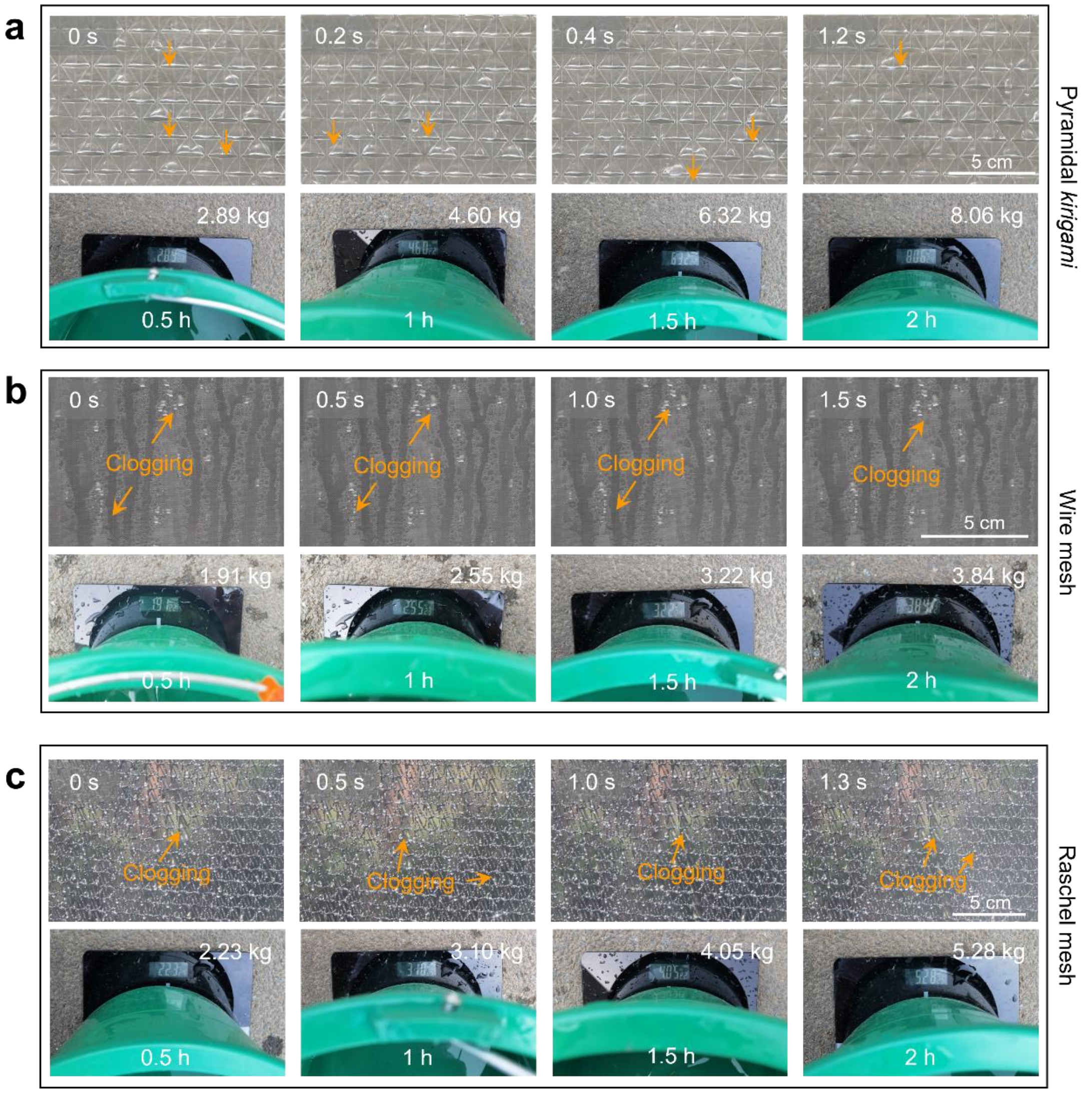

Supplementary Fig. 21| The outdoor testing of fog growth (top panel) and water collection (bottom panel) on meter-sized fog collectors. a, Pyramidal *kirigami* (w_o = 1 cm; θ = 150°). **b**, Wire mesh (wire diameter: 150 μm, open aera: 30%). **c**, Raschel mesh (wire width: 2 mm, shade rate: 50%-60%).

a

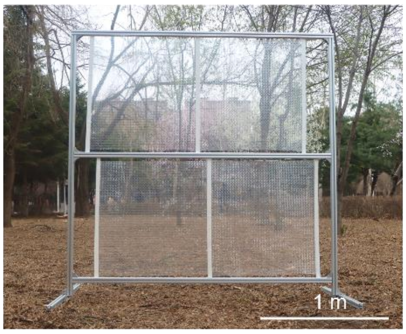

b

	Price (for 4 m^2)	Total price per m^2
Material cost (PET sheet)	\$4-\$45	\$35-\$175
Sample fabrication	\$40-\$120	
Aluminum profile	\$85-\$480	
Connector	\$5-\$30	
Acrylic frame	\$7-\$15	
Other accessories (*e.g.* nuts and bolts)	\$2-\$10	

Supplementary Fig. 22| The modular assembly of pyramidal *kirigami* structures (θ = 150°). **a**, An example of the modularized 2 × 2 panels. Each panel is cut from a 1 m × 1 m PET sheet, and can be adjusted by lateral stretching. **b**, The estimated cost for installation of the modularized kirigami fog collectors. As the price of materials and fabrication cost vary in different places, here we consider the cost in US, Europe and China in the chart.

Supplementary References

1. Karunakaran, R. G., Lu, C. H., Zhang, Z., Yang, S. Highly transparent superhydrophobic surfaces from the coassembly of nanoparticles (<=100 nm). *Langmuir* **27**, 4594-4602 (2011).
2. Lu, Y., Sathasivam, S., Song, J., Crick, C. R., Carmalt, C.J. and Parkin, I. P. Robust self-cleaning surfaces that function when exposed to either air or oil. *Science* **347**, 1132-1135 (2015).
3. Park, K.-C., Chhatre, S. S., Srinivasan, S., Cohen, R. E. & McKinley, G. H. Optimal design of permeable fiber network structures for fog harvesting. *Langmuir* **29**, 13269-13277 (2013).
4. Shi, W., Anderson, M. J., Tulkoff, J. B., Kennedy, B. S. & Boreyko, J. B. Fog harvesting with harps. *ACS Appl. Mater. Interfaces.* **10**, 11979-11986 (2018).
5. Bai, H. *et al.* Cactus kirigami for efficient fog harvesting: Simplifying a 3D cactus into 2D paper art. *J. Mater. Chem. A* **8**, 13452-13458 (2020).
