# Supplementary material for: Aerodynamics-assisted, efficient and scalable kirigami fog collectors
Source: Nat Commun. 2021 Sep 16;12:5484. doi: 10.1038/s41467-021-25764-4 (PMC8445985; doi:10.1038/s41467-021-25764-4)
Supplement: Supplementary file 2 — Description of Additional Supplementary Files [file 41467_2021_25764_MOESM2_ESM.pdf]

Description of Additional Supplementary Files

File Name: Supplementary Movie 1
Description: **PIV characterization revealing the formation of counter-rotating vortex pair around the macroscopic curvature.** Tailored by the global geometric curvature, quasi-stable vortices form in front of the folded substrates, with their position closer to the surface as θ decreases. The existence of vortices can attract and rectify the dynamics of incoming fog droplets, potentially leading to effective fog interception.

File Name: Supplementary Movie 2
Description: **Vertical view of the dynamics of fog water on the cubic kirigami structure versus that on the perforated surface without folding.** There displays pronounced increasement in the droplet growth at the center of cubic kirigami, though the unit size is four orders of magnitude larger than the incoming fog droplet. Here, θ = 150°.

File Name: Supplementary Movie 3
Description: **The continuous water dripping and collection on pyramidal *kirigami* structure.** The conjunction of the aerodynamics-assisted fog capture provided by the geometric curvature and the directed transport enabled by the asymmetric surface morphology allows us to achieve continuous fog collection, circumventing the trade-off between droplet deposition and transport in the conventional design. Here, θ = 150°.

File Name: Supplementary Movie 4
Description: **The high fog collection of pyramidal arrays.** The *kirigami* structure consisting of concave and convex pyramids naturally decouples the multistage fog collection process into different planes, which avoids the clogging problem and lead to higher durability. Here, θ = 150°.

File Name: Supplementary Movie 5
Description: **Outdoor testing of the meter-scale kirigami fog collector.** The meterscale *kirigami* sample displays a fast collection and dripping of fog in the outdoor environment. Here, θ = 150°.
